# Differential Expression of PD-L1 during Cell Cycle Progression of Head and Neck Squamous Cell Carcinoma

**DOI:** 10.3390/ijms222313087

**Published:** 2021-12-03

**Authors:** Daniela Schulz, Martin Wetzel, Jonas Eichberger, Gerhard Piendl, Gero Brockhoff, Anja K. Wege, Torsten E. Reichert, Tobias Ettl, Richard J. Bauer

**Affiliations:** 1 Department of Oral and Maxillofacial Surgery, University Hospital Regensburg, 93053 Regensburg, Germany; daniela.schulz@ukr.de (D.S.); mkwetzel@web.de (M.W.); jonas.eichberger@web.de (J.E.); torsten.reichert@ukr.de (T.E.R.); tobias.ettl@ukr.de (T.E.); 2 Department of Oral and Maxillofacial Surgery, Center for Medical Biotechnology, University Hospital Regensburg, 93053 Regensburg, Germany; 3 Department of Gynecology and Obstetrics, Medical Center Regensburg, 93053 Regensburg, Germany; gerhard.piendl@ukr.de (G.P.); gero.brockhoff@ukr.de (G.B.); anja.wege@ukr.de (A.K.W.)

**Keywords:** PD-L1, HNSCC, head and neck, cell cycle inhibition/blockade/progression, palbociclib, aphidicolin, nocodazole, CDK4/CDK6 inhibitor, epithelial/mesenchymal differentiation status

## Abstract

The expression of PD-L1 by tumor cells is mainly associated with its immunosuppressive effect. In fact, PD-1/PD-L1 immune checkpoint inhibitors demonstrated remarkable effects in advanced cancer patients including HNSCC. In this context, irradiation is currently being investigated as a synergistic treatment modality to immunotherapy. However, the majority of HNSCC patients still show little improvement or even hyperprogression. Interestingly, there is increasing evidence for additional cell-intrinsic functions of PD-L1 in tumor cells. In previous studies, we showed that PD-L1 has a strong influence on proliferation, migration, invasion, and survival after irradiation. We demonstrated that cellular expression and localization of PD-L1 differed depending on sensitivity to irradiation. Here, we show that PD-L1 is also differentially expressed during cell cycle progression of HNSCC. Furthermore, cellular localization of PD-L1 also changes depending on a particular cell cycle phase. Moreover, distinct observations occurred depending on the general differentiation status. Overall, the function of PD-L1 cannot be generalized. Rather, it depends on the differentiation status and microenvironment. PD-L1 expression and localization are variable, depending on different factors. These findings may provide insight into why differential response to PD-1/PD-L1 antibody therapy can occur. Detailed understanding of cell-intrinsic PD-L1 functions will further allow antibody-based immunotherapy to be optimized.

## 1. Introduction

Head and neck squamous cell carcinoma (HNSCC) represents the eighth most common cancer worldwide. Nearly 800,000 new diagnoses are made annually with a large increase in incidence occurring over the past 10 years [1]. A multidisciplinary approach involving surgery, chemotherapy, and radiotherapy is used to treat advanced HNSCC. Despite remarkable progress over the last decades, the 5 year overall survival rate of patients with HNSCC is still approximately 50% [2].

Recently, immunotherapy by inhibition of checkpoint regulators has emerged as an important part of successful treatment. The PD-1/PD-L1 immune checkpoint plays a critical role in regulating T-cell activity during the inflammatory response to infection and controlling autoimmunity [3]. Usually, PD-L1 binds to its receptor PD-1, resulting in attenuation of T-cell activity [4]. PD-L1 is expressed by many cell types, including T cells, B cells, monocytes, APC, and epithelial cells and is upregulated in response to proinflammatory cytokines such as interferon-gamma [5]. Studies show that cancer cells expressing PD-L1 can evade the immune response. Many solid human cancers have been found to express PD-L1, including colorectal cancer, gastric cancer, esophageal cancer, hepatocellular carcinoma, melanoma, glioblastoma, lung cancer, and oral squamous cell carcinoma [6,7,8,9]. Current studies are focused mainly on the immunogenic function of a PD-1/PD-L1 interaction. Early clinical trials in patients with recurrent or metastatic HNSCC—who previously had low prospects of recovery after progression of platinum-based chemotherapy — showed impressive clinical outcomes with the use of the anti-PD-1 antibodies nivolumab or pembrolizumab [10,11]. However, although immune checkpoint inhibition has shown promising results, a substantial number of patients still show little improvement or even hyperprogression after treatment with PD-1/PD-L1 antibodies. Many cancer patients fail to respond to anti-PD-1/PD-L1 treatment, and the underlying mechanisms are not well understood [12,13]. Heterogeneity of PD-L1 expression is common and accounts, to some extent, for some non-predictable results of PD-L1 status [14].

Earlier studies have shown that response to PD-1/PD-L1 blockade may correlate with PD-L1 expression in tumor cells [15,16]. A growing number of publications indicate that PD-L1 has important functions during cell division and the cell cycle. Yu et al. reported PD-L1 involvement during the separation of sister chromatids [17]. Recently, CDK4/6 inhibition was shown to affect the expression levels of PD-L1 in mouse embryonic fibroblasts (MEFs), where a knockout for cyclin D1 revealed an increased level of PD-L. The authors reported that PD-L1 protein levels are regulated by the cyclin D/CDK4/Cullin3-SPOP axis, which regulates PD-L1 protein stability. Cyclin D1-CDK4 directly phosphorylates SPOP at Ser6, which serves as an adaptor protein for Cullin3 ubiquitin ligase and promotes proteasome-mediated PD-L1 degradation [18]. Thus, there is increasing evidence that PD-L1, in addition to its immunoregulatory effect, has some cell-intrinsic functions that may affect particular tumor-associated properties [19,20,21,22]. In previous studies, we demonstrated the strong influence of PD-L1 on proliferation, migration, invasion, and survival after irradiation [23]. Furthermore, we revealed differential expression and localization of PD-L1 depending on the sensitivity to irradiation [24].

To obtain deeper insight into PD-L1 expression during cell cycle progression in HNSCC, we investigated whether PD-L1 levels in HNSCC cell lines were associated with different cell cycle phases. We show that PD-L1 expression during cell cycle progression in HNSCC cells varies depending on the specific cell cycle phase and whether the cells exhibit epithelial or mesenchymal properties. Moreover, the cellular localization of PD-L1 also changes depending on a particular cell cycle phase. In addition, different observations were made depending on the general differentiation status. It has been shown that membrane-bound PD-L1, which is considered a target of successful immunotherapy, is mainly presented in the proliferative status, more specifically in the S and partially in the G2/M phase. Overall, our findings may shed light on why differential response to PD-1/PD-L1 antibody therapy may occur. A detailed understanding of the cellular functions and complex regulation of PD-L1 will allow further optimization of antibody-based immunotherapy.

## 2. Results

**Figure 1:**
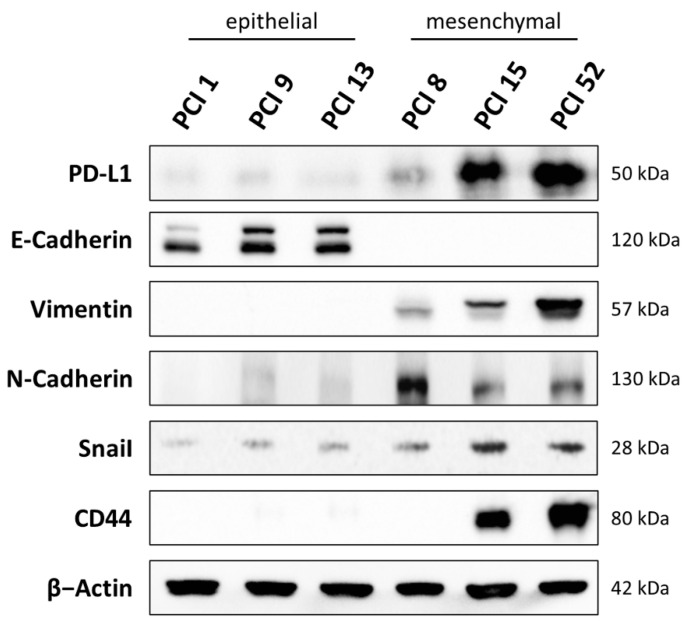
Characterization of HNSCC cell lines. The basal expression of PD-L1, E-cadherin, vimentin, N-cadherin, CD44, and Snai1 of the HNSCC cell lines PCI 1, 9, 13, 8, 15, and 52 were investigated in a WB analysis. The cells were harvested in a sub-confluent condition. β-actin served as a loading control. Thirty milligrams of total protein lysate were used. Based on these markers, cells were divided into epithelial and mesenchymal differentiation status. Original western blots in Supplementary Materials S1.

For the experiments, HNSCC cell lines PCI 1, 9, 13, 8, 15, and 52 were characterized. In Figure 1, basal expression of PD-L1, the epithelial and mesenchymal differentiation markers E-cadherin, vimentin, and N-cadherin, the HNSCC tumor stem cell marker CD44 as well as the oncogenic transcription factor Snai1 was determined using WB analysis. Based on these markers, the cell lines were assigned to either an epithelial or a mesenchymal differentiation status.

The HNSCC cell lines PCI 1, 9, and 13 displayed an epithelial character. In these cell lines, a strong expression of the epithelial marker E-cadherin, promoting cell-to-cell contact and a weak or absent expression of vimentin, N-cadherin, Snai1, and CD44 were detected. These cell lines also showed low PD-L1 expression.

In contrast, the HNSCC cell lines PCI 8, 15, and 52 were assigned to a mesenchymal differentiation status. They showed a strong expression of the mesenchymal marker vimentin and N-cadherin, promoting cell migration and metastasis as well as the HNSCC tumor stem cell marker CD44 and overexpression of the oncogenic transcription factor Snai1. These cell lines also showed moderate to high PD-L1 expression compared to the epithelial HNSCC cell lines. PCI 8, originating from a metastasis, was not always clear to assign. PCI 8 showed the highest expression of the mesenchymal marker N-cadherin in the absence of E-cadherin, yet it did not express CD44 and exhibited only a slight expression of the transcription factor Snai1 and the mesenchymal marker vimentin.

**Figure 2:**
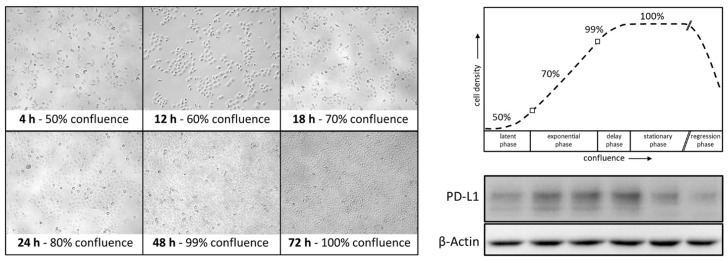
Confluence-dependent expression of PD-L1. HNSCC cell lines were analyzed 4, 12, 18, 24, 48, and 72 h after seeding. Depending on the incubation time and/or cell density, which was recorded by photo documentation at 20-fold magnification, different PD-L1 expression levels could be detected. Here, cell line PCI 9 is shown as a representative example of the WB analysis. β-actin served as a loading control.

Interestingly, confluence-dependent expression of PD-L1 was observed in HNSCC cell lines. Figure 2 shows exemplified PCI 9 cells at different confluence stages 4, 12, 18, 24, 48, and 72 h after seeding. Cells were seeded with a confluence of approximately 50% and reached 100% after 72 h cell culture. Cell density is shown graphically with corresponding PD-L1 expression in a representative WB analysis. PD-L1 expression increased proportionally with increasing confluence. At 99–100% confluence, between 48 and 72 h of growth, PD-L1 immunodetection decreased again with time.

To further investigate whether PD-L1 expression is cell cycle dependent, cell cycle arrest was performed at distinct cell cycle phases. The cell cycle was blocked in the G1, S, and G2/M phases using palbociclib, aphidicolin, and nocodazole, respectively. For each cell line, the effective inhibitor concentrations were determined separately. Initially, inhibitor concentrations were approximated using MTT viability assays. IC_50_ values were determined 48 h after inhibitor treatment. The precise effective concentrations were subsequently determined by flow cytometry. FACS analyses were performed to determine the balance between efficacy and toxicity of the inhibitor for each cell line individually.

**Figure 3:**
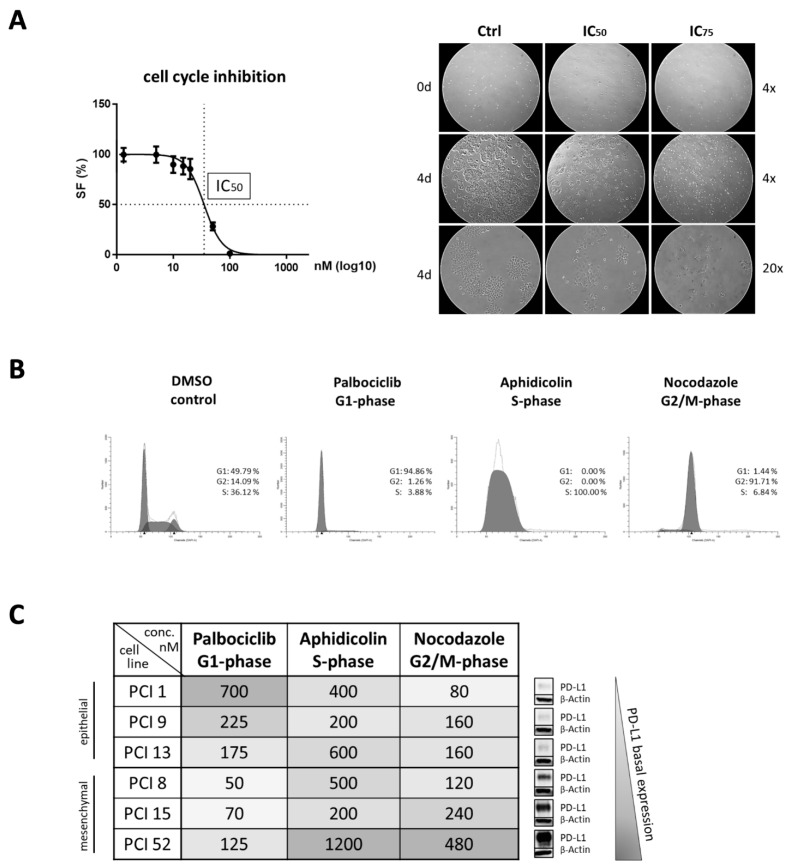
Efficacy of cell cycle inhibitors. (**A**) Inhibition of cell proliferation due to the incubation with cell cycle inhibitors. An MTT viability assay was used for IC_50_ determination. Here, PCI 1 was chosen as the representative cell line with nocodazole (G2/M phase) treatment. Photo documentation after four days of inhibitor treatment confirmed the inhibition of cell proliferation. PCI 13 was chosen as the representative cell line with aphidicolin (S phase) treatment. Images are shown at four- and 20-fold magnification. (**B**) Cell cycle analysis via flow cytometry after inhibitor treatment. Flow cytometry confirmed that palbociclib treatment resulted in G1 phase, aphidicolin in S phase, and nocodazole in G2/M phase arrest. Here, PCI 13 was also chosen as the representative cell line. Incubation with DMSO instead of a specific inhibitor were used as controls. To quantify the DNA content, cells were incubated with DAPI. For analysis 5 × 10^4^ cells were used per sample. The proportion of cells in a particular cell cycle phase is expressed as a percentage (%). (**C**) Effective concentrations of cell cycle inhibitors. Concentrations for palbociclib, aphidicolin, and nocodazole are listed in nanomolar (nM). High concentrations are highlighted in dark, whereas low concentrations are highlighted in light colors. Cell lines were subdivided into their epithelial and mesenchymal differentiation status and ranked according to their PD-L1 basal expression.

Figure 3A shows an example of the IC_50_ values determined with the PCI 1 cell line after 48 h of incubation with nocodazole. Photo documentation after four days of inhibitor treatment with aphidicolin confirmed the inhibition of cell proliferation in each cell line. Cell line PCI 1 was chosen as a representative.

Flow cytometric cell cycle analyses confirmed all three inhibitors—palbociclib, aphidicolin, and nocodazole—successfully arrested the cell cycle at the correct phase. Histograms, as shown in Figure 3B, verified an arrest in the G1 phase with palbociclib, in the S phase with aphidicolin, and in the G2/M phase with nocodazole. Here, PCI 13 was selected as a representative cell line. Effective inhibitor treatment at the correct cell cycle phase was determined separately for all HNSCC cell lines. As a control, cells were incubated with the solvent DMSO. The samples treated with DMSO represent a mixed cell population, with randomly distributed cells in different cell cycle phases. This flow cytometric analysis also served to determine the appropriate inhibitor concentrations that most effectively inhibited the cell cycle phase.

HNSCC cell lines with different PD-L1 basal expressions were used for our experiments. PCI 1, 9, and 13 have very low PD-L1 expression, whereas PCI 8, 15, and 52 have moderate to high expression. Interestingly, basal expression of PD-L1 also appeared to be correlated with the response to the cell cycle inhibitors as shown in Figure 3C. Cell lines with epithelial characteristics as well as with very low PD-L1 expression required up to 10-fold higher inhibitor concentration of palbociclib compared to cells with high PD-L1 expression. Conversely, cells with mesenchymal characteristics and moderate to high PD-L1 levels required 2–6-fold higher concentrations of aphidicolin and nocodazole to block the cell cycle effectively.

To determine PD-L1 expression during cell cycle progression, we performed WB analysis of PD-L1. To support these observations, we additionally performed cytochemical staining of PD-L1 with DAB. In advance, HNSCC cell lines PCI 1, 9, 13, 8, 15, and 52 were specifically blocked at the cell cycle phases G1, S, or G2/M with the cell cycle inhibitors palbociclib, aphidicolin, or nocodazole. DMSO-treated cells served as controls. They depict PD-L1 expression of a mixed cell population of randomly distributed cells in different cell cycle phases.

**Figure 4:**
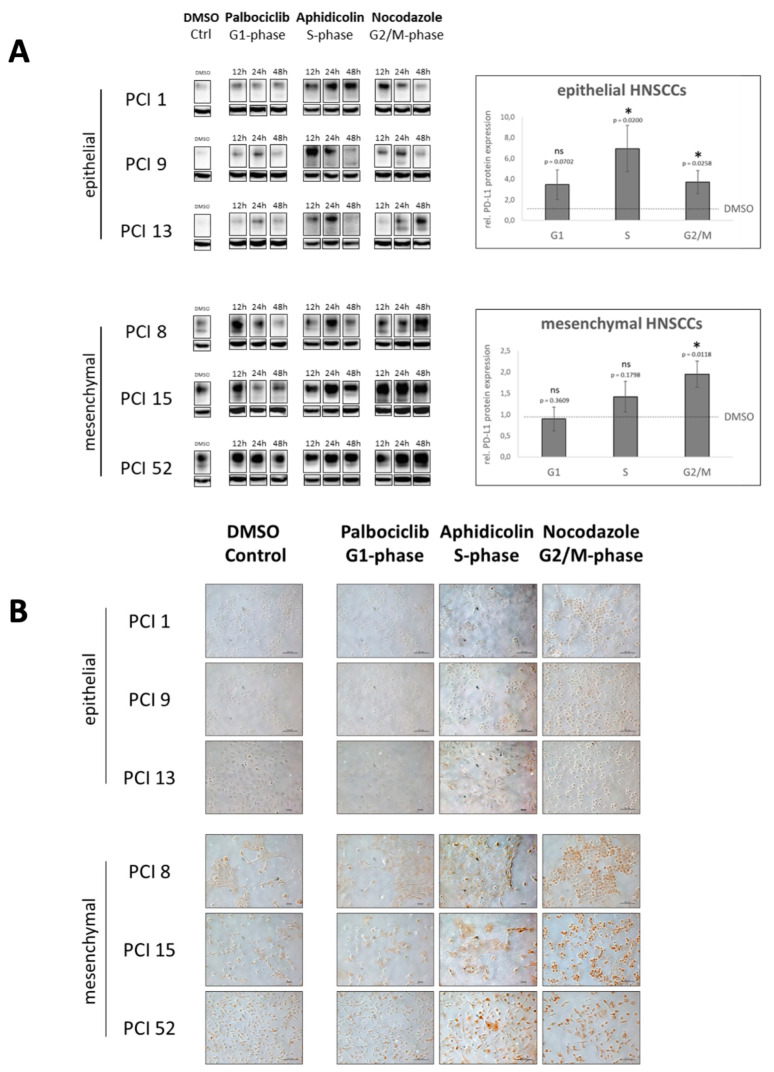
PD-L1 expression during cell cycle progression. HNSCC cell lines PCI 1, 9, 13, 8, 15, and 52 were specifically blocked during cell cycle phases G1, S, or G2/M. DMSO-treated controls showed PD-L1 expression of a mixed cell population of randomly distributed cells in different phases. (**A**) WB and semiquantitative analysis after inhibitor treatment in a time-dependent manner for 12, 24, and 48 h. β-actin served as a loading control. For statistical analysis, the DMSO control of the respective cell line was chosen as a reference (dashed line). The results are expressed as means ± SD (standard deviation). *n* = 3. Paired *t*-test (ns = *p* ≥ 0.05; * = *p* < 0.05). (**B**) Immunocytochemical DAB staining of PD-L1. Representative images are shown at 20-fold magnification. The scale represents 0.1 mm. Expression of PD-L1 is visible as brown staining. In Supplementary Materials Figure S2, incubation with an IgG isotype control demonstrates the specificity of PD-L1 staining.

In Figure 4A, WB analysis reveals a difference in the PD-L1 expression pattern depending on whether HNSCC cells had epithelial or mesenchymal characteristics. In cells with epithelial characteristics (i.e., PCI 1, 9, and 13), PD-L1 expression increased in a growth time-dependent manner, especially after inhibition of the S phase with aphidicolin.

However, cells with mesenchymal characteristics (i.e., PCI 8, 15, and 52), exhibited increased PD-L1 expression or accumulation after blockade in both the S phase and the G2/M phase in a time-dependent manner. Compared with the DMSO control, PD-L1 expression increased in the cells with epithelial features at each phase after cell cycle arrest. Cells with mesenchymal features showed accumulation of PD-L1 only in the S and especially in the G2/M phase of the cell cycle.

To confirm the observations of the WB analysis, DAB staining was performed. Representative images are shown in Figure 4B. Cells were blocked in the G1, S, and G2/M phases according to cell cycle inhibitor-dependent blocking times. The staining confirmed the WB results. The HNSCC cell lines showed different PD-L1 expression levels depending on both the blocked cell cycle phase and the epithelial–mesenchymal characteristics of the cells.

In both cell types, it is clearly visible that PD-L1 expression was induced during the S phase compared with the DMSO control and G1 phase inhibition with palbociclib, which is indicated by a distinct brown staining. During the S phase, cells of all HNSCC cell lines increased significantly in size.

In the G2/M phase, a significant increase in PD-L1 expression was observed, especially in the cell lines with mesenchymal features, but also in the cell lines with epithelial features. In the G2/M phase, the cells were significantly smaller and had a round shape. In the M phase, they barely attached to the cell culture dish. Specifically, these cells had a very pronounced brown staining. The exact cellular localization of PD-L1 could not be clearly determined at this point.

Since the success of antibody-based therapy may depend on the presence of the target molecule on the membrane, we specifically examined the membrane expression of PD-L1 in relation to the cell cycle phase into which the cell has entered. For this purpose, flow cytometric analyses were performed.

**Figure 5:**
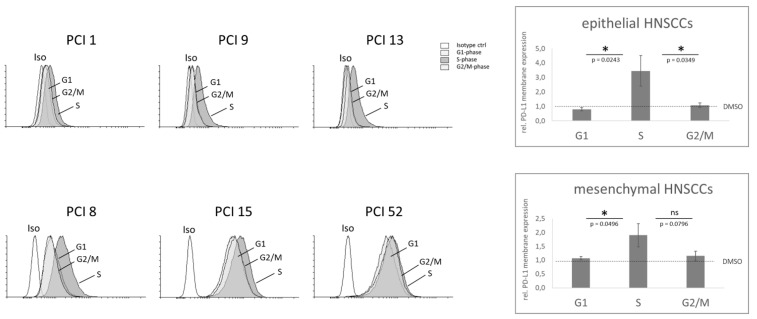
PD-L1 membrane expression during cell cycle progression. Cell cycle progression of the HNSCC cell lines PCI 1, 9, 13, 8, 15, and 52 were blocked during the G1 (grey), S (grey), or G2/M phase (grey). Cell cycle analysis via flow cytometry was performed using flow cytometry. To quantify the DNA content, cells were incubated with DAPI. 5 × 10^4^ cells were used per sample. Histograms (left) indicate PD-L1 membrane expression of each single HNSCC cell line. An IgG2κ isotype antibody (white) was used as the negative control. For signal quantification (right), the three epithelial cell lines (i.e., PCI 1, 9, and 13) and the three mesenchymal cell lines (i.e., PCI 8, 15, and 52) were combined. The DMSO-treated sample of the respective cell line was chosen as the reference (dashed line). The results are expressed as the means ± SD (standard deviation). *n* = 3, paired *t*-test (ns = *p* ≥ 0.05; * = *p* < 0.05).

Interestingly, Figure 5 shows that all the examined HNSCC cell lines exhibited a significant increase in PD-L1 expression on the cell membrane after S phase arrest, implying a role of PD-L1 in this phase. PCI 52, with mesenchymal characteristics and the highest PD-L1 basal expression of all examined HNSCC cell lines, was the only one of all the cell lines examined that also showed an increase in PD-L1 membrane expression in the G2/M phase.

Semiquantitative analysis revealed that in both epithelial and mesenchymal HNSCC cell lines, S phase arrest resulted in significantly higher PD-L1 membrane expression than G1 phase. There was no significant difference in PD-L1 membrane expression between the G1 and G2/M phase in all HNSCC cell lines. There was also a significant difference between the S phase and G2/M phase in the HNSCC cell lines with epithelial characteristics (i.e., PCI 1, 9, and 13). In the HNSCC cell lines with mesenchymal characteristics (i.e., PCI 8, 15, and 52), the significant difference between the S and G2/M phase was attenuated by the increase in the PD-L1 membrane expression of the cell line PCI 52.

To determine whether PD-L1 itself has an impact on cell cycle progression, we reduced PD-L1 expression via siRNA knockdown and subsequently quantified the proportion of cells in distinct cell cycle phases via FACS analysis. Control cells were transfected with non-targeting siRNA (NT), and the results are given in comparison to NT controls.

**Figure 6:**
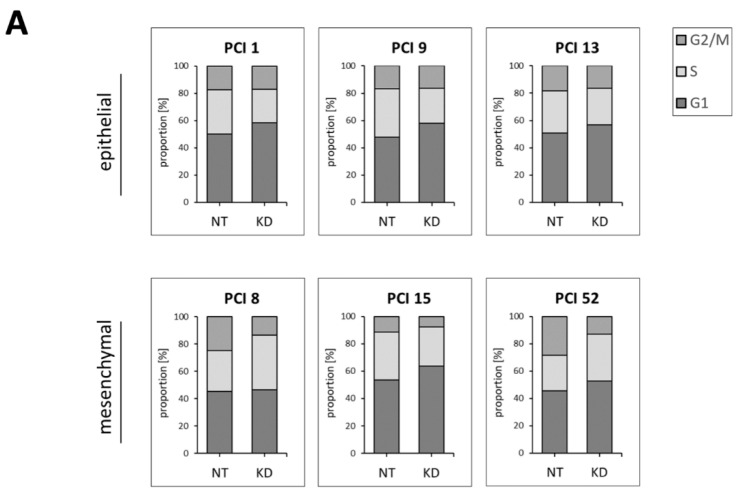

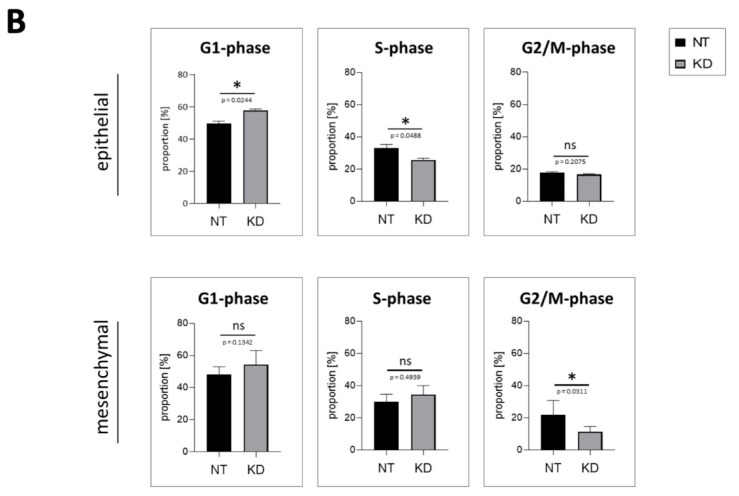
Proportion of specific cell cycle phases after PD-L1 knockdown. In the six different HNSCC cell lines (i.e., PCI 1, 9, 13, 8, 15, and 52), PD-L1 levels were reduced after siRNA knockdown (KD). Transfection with non-targeting siRNA (NT) served as the control. (**A**) Stacked bar charts show the percentage of cells in the G1, S, or G2/M phase. All three phases together sum up to 100%. (**B**) For statistical analysis, PD-L1 KD samples of the three epithelial and three mesenchymal cell lines were compared with their respective NT controls, using ratio paired *t*-tests (ns = *p* ≥ 0.05; * = *p* < 0.05). *n* = 3. KD efficiency is shown in Supplementary Materials Figure S3. The effect on the proliferation rate of epithelial HNSCCs is shown in Supplementary Materials Figure S4, while the effect on mesenchymal HNSCCs is shown in Schulz et al. Figure 5 [25].

Figure 6 shows that lowering levels of PD-L1 impaired cell cycle progression. This observation differed between HNSCC cells with epithelial and mesenchymal characteristics, respectively. In cell lines with epithelial characteristics (i.e., PCI 1, 9, and 13), PD-L1 KD resulted in a significant decrease of cells in the S phase favoring the G1 phase. The G2/M phase remained unaffected. In contrast, in cell lines with mesenchymal characteristics (i.e., PCI 8, 15, and 52), PD-L1 KD resulted in a significant decrease of cells in the G2/M phase favoring the G1 and S phases. In Figure 6A, cell cycle phases G1, S, and G2/M are presented combined in stacked bar charts. Here, the result of each individual cell line is presented. In Figure 6B, the same data are presented separately for each cell cycle phase. Here, the three HNSCC cell lines with epithelial characteristics and the three HNSCC cell lines with mesenchymal characteristics are plotted.

## 3. Discussion

Basically, HNSCC is a tumor type that is highly immunogenic due to the high number of somatic mutations. [26]. HNSCC tumors therefore produce many neoantigens and, if HPV infection is involved, they are potentially also targeted by T cells due to the viral antigen expression thereby provoking an immune response [27]. However, a wide variety of studies showed that this initially strong immunogenicity is largely suppressed [28]. Blockade of the immune checkpoint PD-1/PD-L1 showed great results in preventing tumor escape and inhibiting tumor growth by restoring the cytotoxicity of T cells and NK cells [29] and reducing the population and activity of MDSCs and Tregs [30].

In 40––70% of all HNSCC cases, a relatively high expression of PD-L1 has been observed. This is accompanied by upregulation of PD-1 on the majority of CD8+ tumor-infiltrating lymphocytes (TILs) [31,32]. Studies also demonstrated that PD-1 expression increased in samples from patients with HNSCC compared with normal oral mucosa samples [33], suggesting that PD-1 blockade should actually be more effective in enhancing the antitumor immune response in HNSCC. However, data from recent clinical trials showed an overall moderate response rate (ORR) to PD-1 blockade of less than 20% [10,34] and a lack of marked responses in most patients [35], compared with more impressive ORRs of up to 57% in other advanced/pretreated tumor types such as non-small cell lung cancer and melanoma [36,37]. Therefore, only a small number of patients with HNSCC may benefit from PD-1/PD-L1 blockade. In our studies, we explored the underlying reason behind these findings. In recent years, a growing number of studies have concluded that PD-L1 may not only have immunogenic but at the same time cell-intrinsic functions [23,24,38]. This study was conducted because we observed a confluence- and time-dependent fluctuation of PD-L1 expression in all our HNSCC cell lines.

As shown in Figure 1, we initially observed confluence- and time-dependent PD-L1 expression in all HNSCC cell lines examined. Once the cells entered the exponential proliferation phase under optimal culture conditions, PD-L1 expression was at its peak. When cells were restrained from proliferation due to the lack of space and increasing cell–cell contact (48–72 h at a 99–100% confluence), PD-L1 expression simultaneously decreased. Tufano et al. reported a fluctuation of PD-L1 expression concurrently with cyclin D in glioblastoma cells. Consistent with our findings, they detected an increase in PD-L1 very early after cell seeding, which was accompanied by the increase in cyclin D expression and gradually decreased as cell growth progressed until cell confluence, where the study registered the lowest level, identical to what we observed in our HNSCC cell lines [39]. This intrigued us to determine whether PD-L1 is more abundant at certain cell cycle phases compared to others in HNSCC cells.

To investigate this hypothesis, our goal was to create an enriched cell population at a discrete phase of the cell cycle. After removal of the inhibitor, these cells should then be able to continue the cell cycle with minimal impairment of regular functions. Therefore, we put great emphasis on determining the effective concentration and incubation time of each inhibitor used to minimize toxic side effects. We observed a more than four-fold increase in PD-L1 expression after palbociclib treatment compared to DMSO control in HNSCC cells with epithelial features but not in cells with mesenchymal characteristics. Recent clinical studies have shown that monotherapies with palbociclib have not been exceptionally successful. The increase of PD-L1 in HNSCC cells with certain characteristics after treatment with palbociclib and arrest in the G1 phase may contribute to a lack of therapeutic response due to the intrinsic carcinogenic mechanisms of PD-L1 overriding cell inhibition. In line with our results demonstrating PD-L1 increase after CDK4/6 inhibition, several studies report PD-L1 level elevation after CDK4/6 inhibition or pRB depletion [40]. Furthermore, a study by Zhang et al. describes destabilization of PD-L1 by cyclin D/CDK4/6 kinase via proteasome-mediated degradation. Inhibition of CDK4 and CDK6 in vivo increases PD-L1 protein levels by interfering with cyclin D-CDK4-mediated phosphorylation of speckle-type POZ protein (SPOP), resulting in the degradation of SPOP by the anaphase-promoting complex activator FZR1 [18].

Furthermore, we detected a significant increase in total PD-L1 protein expression in the G2/M phase after cell cycle arrest with nocodazole in all HNSCC cell lines with epithelial and mesenchymal characteristics, with mesenchymal cells showing the highest PD-L1 levels in the G2/M phase. In line with these data, in ovarian cancer and chronic myeloid leukemia cells’ G2 phase arrest with a CDK-1 inhibitor resulted in the highest PD-L1 gene expression. Moreover, M phase arrest with nocodazole resulted in the highest PD-L1 protein expression compared with G1 cell cycle arrest. Hereby, the expression was non-uniform during mitosis but changed at different mitotic stages, with PD-L1 levels increasing in cytokinesis compared with mitotic phase [41]. Yu et al. reported PD-L1 involvement during the separation of sister chromatids [17]. They described that PD-L1 functions as a subunit of the cohesin complex. In this context, deficiency of PD-L1 leads to the formation of multinucleated cells and thereby causes a defect in sister chromatid cohesion. Mechanistically, PDL1 compensates for the loss of sororin, the expression of which is suppressed in cancer cells overexpressing PD-L1. PD-L1 competes with wing apart-like (WAPL) for binding to PDS5B and ensures proper sister chromatid cohesion and segregation. Zhang et al. found a regulatory mechanism in pancreatic cancer that actively stabilizes PD-L1 in this particular cell cycle phase and protects it from degradation by the proteasome. They found that PD-L1 is a substrate of NEK2 (never in mitosis gene A (NIMA)-related kinase 2). NEK2 is a multifunctional protein physiologically involved in cell cycle regulation such as centrosome duplication and separation, microtubule stabilization, kinetochore attachment, and spindle assembly checkpoint. NEK2 interacts with PD-L1, phosphorylates the T194/T210 residues, and prevents the degradation of PD-L1 in the ER lumen mediated by the ubiquitin–proteasome pathway. Inhibition of NEK2 thereby sensitizes PD-L1 blockade and synergistically enhances the immune response against pancreatic cancer. Combinatorial inhibition of NEK2 and PD-L1 significantly improves therapeutic efficacy in pancreatic cancer in preclinical models [42].

We also examined PD-L1 membrane expression during different cell cycle phases, as membrane-bound PD-L1 in particular serves as a target for antibody-based immunotherapy. Most interestingly, PD-L1 membrane expression, both in cells with epithelial and mesenchymal characteristics, was highest after S phase arrest, respectively. As in the analysis of total protein expression, cells with epithelial features exhibited the greatest changes in PD-L1 expression in S phase arrest. Since PD-L1 total and membrane expression increased in the proliferative state of all investigated HNSCC cell lines, it seems important to discuss suitable combination therapies for future applications.

To investigate whether PD-L1 is essential for cell cycle progression of HNSCCs at some point, PD-L1 knockdown was performed in all HNSCC cell lines. It was of interest to determine whether downregulation of PD-L1 ultimately led to an impediment of cell cycle progression. In our previous work, we already demonstrated for the mesenchymal HNSCC cell lines PCI 8, 15, and 52 that PD-L1 KD leads to a significant reduction of proliferation [25]. Here, we also demonstrated a reduction in proliferation after PD-L1 KD in epithelial HNSCCs but to a lesser extent (Supplementary Materials Figure S4). Consistently, PD-L1 knockdown using siRNA significantly decreased the numbers of cells that were in the S phase in all cells with epithelial characteristics, whereas two out of three mesenchymal cell lines showed an increase in the S phase. This implies that the G1/S transition fails in epithelial HNSCCs lacking PD-L1. Cells accumulate in G1, while the proportion of cells in the S phase decreases significantly. Since we did not observe accumulation of cells in the G2/M phase, this suggests that they were arrested in this cell cycle phase. Cells might not accumulate in this phase because of a lack of cell replenishment. Parkes et al. identified a molecular subtype of breast cancer in which the DNA damage response is deficient (DDRD). They found that DDRD breast tumors were associated with infiltration of CD4+ and CD8+ lymphocytes. DDRD cells exhibited increased cytosolic DNA and constitutive activation of the cGAS/STING/TBK1/IRF3 pathway for viral response. Importantly, this pathway was activated in a cell cycle-specific manner. They showed that DNA damage in the S phase activates the expression of PD-L1. Activation of this pathway and associated PD-L1 expression may explain the paradoxical lack of T-cell-mediated cytotoxicity observed in DDRD tumors [43]. In mesenchymal HNSCCs, cells without PD-L1 remain in the G1 and S phase but fail to transition into the G2/M phase. As the G2/M phase decreases, these cells seem to be able to complete division. Zoe et al. also reported a correlation between PD-L1 reduction and inhibition of S phase progression. In their research, curcumol inhibited cell proliferation, S phase progression, tube formation, invasion, and metastasis by inhibiting PD-L1. Moreover, curcumol restored the activity of cytotoxic T cells and their ability to kill tumor cells by inhibiting PD-L1. Curcumol inhibited the expression of PD-L1 by overlapping the HIF-1α and p-STAT3 (T705) signaling pathways in liver cancer [44].

In summary, our data clearly indicate an important cell-intrinsic function of PD-L1 in cell cycle control in HNSCC cells. This might also be a reason for the significantly lower therapeutic response to antibody therapy in HNSCC patients. A precise determination of the individual characteristics of the tumor tissue prior to treatment seems to be crucial at this point. However, finding suitable markers that correlate with tumor response has been difficult so far. The exact mechanisms of PD-L1 regulation, its fluctuating expression and cellular localization during cell cycle progression are still unclear. In the future, we intend to elucidate the underlying mechanisms.

## 4. Material and Methods

### 4.1. Cell Lines and Culture Conditions

The human HNSCC cell lines PCI 1, PCI 8, PCI 9, PCI 13, PCI 15, and PCI52 were kindly provided by Theresa. L. Whiteside (University of Pittsburgh Cancer Institute (PCI), Pittsburgh, PA, USA) in 2013. The cell lines were established from primary tumors of different origin in the laboratory at the University of Pittsburgh: PCI 1—larynx, PCI 8—pyriform sinus, PCI 9—base of tongue, PCI 13—retromolar triangle, PCI 15—pyriform sinus, and PCI 52—plica aryepiglottica [45,46]. HNSCC cell lines were maintained in DMEM (PanBiotech, Aidenbach, D) supplemented with 10% fetal calf serum (FCS, Gibco, Carlsbad, CA, USA), 1% L-glutamine (Sigma–Aldrich, St. Louis, MO, USA), and 1% penicillin/streptomycin (Sigma–Aldrich) at 37 °C in a 5% CO_2_ humidified atmosphere. The medium was changed every two to three days, and the cells were passaged prior to reaching confluence. Detachment of the cells was achieved by incubation with 0.05% trypsin–EDTA solution (Sigma–Aldrich) for 5 to 10 min (min) at 37 °C.

### 4.2. Cell Line Authentication

Cell line authentication of the human HNSCC cell lines PCI 1, PCI 8, PCI 9, PCI 13, PCI 15, and PCI 52 was performed by the Leibniz Institute German Collection of Microorganisms and Cell Cultures (DSMZ) via STR-DNA-typing using nonaplex PCR.

### 4.3. Reagents

Palbociclib (PD 0332991 isethionate), a potent cyclin-dependent CDK4 and CDK6 inhibitor that induces G1 cell cycle arrest, was obtained from Tocris Bioscience (Bristol, UK). A 10 mM stock solution was prepared in dimethyl sulfoxide (DMSO) and stored in aliquots at −20 °C.

Aphidicolin, a specific inhibitor of DNA polymerase α and δ in eukaryotic cells that blocks the cell cycle at early S phase, was obtained from Merck (Darmstadt, D). A 2.9 mM stock solution was prepared in DMSO and stored in aliquots at −20 °C.

Nocodazole, which interferes with the structure and function of microtubules in interphase and mitotic cells and blocks cell cycle in the G2/M phase, was obtained (Sigma–Aldrich) as 5 mg/mL, ready-made DMSO solution, and stored in aliquots at −20 °C.

Prior to application, dilutions of cell cycle inhibitors were prepared using antibiotic-free medium. Cell cycle inhibition was not performed earlier than 24 h after seeding. Effective inhibitor concentrations were obtained by calculation of the IC_50_ values 48 h after inhibitor treatment. The calculated values were then further adjusted based on cell cycle analyses using flow cytometry. The efficacy of the three used inhibitors—palbociclib, aphidicolin, and nocodazole—was tested for each cell line separately after 12, 24, and 48 h of incubation. The earliest time point that showed an adequate cell cycle inhibition with minimal toxic effects was selected for all further experiments. For palbociclib and nocodazole, this was already the case after 12 h and for aphidicolin after 24 h.

As a control, cell culture medium without inhibitor containing only DMSO was used. The selected DMSO concentration was equal to the concentration of DMSO present in the highest inhibitor concentration.

### 4.4. MTT Cell Viability Assay

For determination of IC_50_ via TACS^®^ MTT Cell Proliferation assay (R&D Systems, Minneapolis, MN, USA), cells were seeded in 96-well plates (Greiner Bio-One, Kremsmünster, AUT). The cell number was adjusted to the proliferation rate of each cell line to ensure that the cells remained sub-confluent until analysis. The number of cells ranged from 800 to 4000 cells/well depending on the cell line and incubation time. The medium was replaced 24 h after seeding with 100 µL medium containing a cell cycle inhibitor. After treatment, the cell proliferation rate was determined according to the manufacturer’s protocol. Ten microliters of MTT reagent were added to each well, and cells were incubated at 37 °C for 120 min. Subsequently, 100 μL detergent reagent was added to each well, and the cells were incubated overnight at RT. Absorbance was measured at 595 nm, and the percent viability was calculated by normalizing the absorbance values to cells grown in medium without inhibitors. For IC_50_ determinations, the inhibitor concentrations tested ranged from 0.1 µM to 30 μM. IC_50_ values 48 h after inhibitor treatment were calculated for each cell line using GraphPad Prism 8 software (GraphPad Software, Inc., San Diego, CA, USA).

### 4.5. Flow Cytometry

Experiments were performed at the FACS Canto II (BD Biosciences, Franklin Lakes, NJ, USA) cytometer with FACSDiva Software 7.0 (BD Biosciences).

For cell cycle analysis cells were seeded in 6-well plates (Greiner Bio-One), and 5 × 10^5^ adherent, sub-confluent cells were detached with Accutase solution (Sigma–Aldrich) for 5–10 min at 37 °C. The single cell suspension was washed properly before fixation. Fixation was performed with 500 µL of 70% methanol in PBS overnight at 4 °C. RNA digestion was conducted with 25 µg Ribonuclease A (Sigma–Aldrich) for 20 min at 37 °C. The quantification of DNA content was performed with 1 µg/mL DAPI (Sigma–Aldrich) for at least 30 min at RT. Cell cycle phases were determined using ModFit LT software 5.0 (Verity Software House).

For determination of PD-L1 membrane expression, cells were seeded as previously described but were kept on ice after detachment during all washing and incubation steps. Viable, non-fixed cells were used for this analysis. Membrane-bound PD-L1 was stained with a phycoerythrin (PE)-conjugated anti-CD274 antibody (clone 29E.2A3, BioLegend, San Diego, CA, USA). The corresponding mouse immunoglobulin IgG2b,κ PE (clone MPC-11, BioLegend) was used as an isotype control, and 5 × 10^4^ cells from each sample were used for analysis. Overlay histograms were generated using Flowing Software 2.5.1 (Turku Centre for Biotechnol, University Turku, FIN).

### 4.6. Protein Isolation

Adherent cells were washed with PBS and then lysed in radioimmunoprecipitation buffer (RIPA) (Sigma–Aldrich) containing protease inhibitors (complete mini-protease inhibitor cocktail) (Roche Diagnostics, Basel, CH). To optimize cell lysis, harvested cells were sonicated on ice for 20 s. Cell pellets and supernatant were separated by centrifugation at 14,000× *g* for 10 min at 4 °C. The supernatant was stored at −20 °C for further analysis.

### 4.7. Western Blot Analysis

For quantitative protein analysis, 30 μg of total protein was used per sample. Protein concentration was determined using a bicinchoninic acid (BCA) assay (Merck). Therefore, proteins were denatured at 70 °C for 10 min in a Laemmli sample buffer (Bio-Rad, Hercules, CA, USA) containing 1% β-mercaptoethanol (Merck). Samples were separated by SDS-PAGE with a 10% dissolution gel and transferred onto a PVDF membrane (Roche). The membrane was blocked with 5% skimmed milk (Carl Roth, Karlsruhe, D) or 3% BSA (bovine serum albumin) Fraction V (Biomol, Hamburg, D) in a TBS buffer containing 0.1% Tween 20 (Sigma–Aldrich) for 1 h at room temperature. Subsequently, the membrane was incubated overnight at 4 °C with a specific primary antibody. Primary antibodies used for WB analysis were anti-PD-L1 (rabbit mAb, clone E1L3N, #13684, Cell Signaling Technology, Danvers, MA, USA (CST)), anti-E-cadherin (mouse mAb, 610182, BD Biosciences), anti-vimentin (rabbit mAb, #5741, CST), anti-N-cadherin (mouse mAb, 610921, BD Biosciences), anti-Snai1 (mouse mAb, sc-393172, Santa Cruz), and anti-CD44 (mouse mAb, #3570, CST). Afterwards, the membrane was additionally incubated with a horseradish peroxidase (HRP)-conjugated secondary antibody. The goat anti-rabbit (#32460, Thermo Scientific, Waltham, MA, USA) and goat anti-mouse antibodies with stabilized peroxidase conjugation (#32430, Thermo Scientific) were used for signal detection. Roti Lumin (Carl Roth) or SuperSignal West Femto Maximum Sensitivity Substrate (Thermo Scientific) was used as substrate. Colorimetric and chemiluminescent images were processed using the high-resolution, high-sensitivity ChemiDoc XRS+ Imaging System (Bio-Rad). For comparison of different membranes, a reference control was used. The reference sample was aliquoted into 30 µg portions immediately after lysis and frozen for storage at −80 °C. The reference control was thawed only once and additionally applied to the corresponding blots. Equal loading of proteins was verified with a specific antibody against β-actin (rabbit pAb, ab8227, Abcam). The housekeeping protein was incubated on the same membrane after stripping with ReBlot Plus Mild Antibody Stripping Solution (Merck). Normalization and quantification were performed using Image Lab software 5.2.1 (Bio-Rad).

### 4.8. Immunocytochemical DAB Staining

For immunocytochemical DAB staining of PD-L1, cells were seeded in 8-chamber slides (BD Biosciences). Cells were fixed in 2% paraformaldehyde (PFA) (minimum 37%) (Merck) for 10 min. Prior to staining, endogenous peroxidase activity was blocked by incubating the cells with 10% H_2_O_2_ (hydrogen peroxide solution) (Sigma–Aldrich) and 10% MeOH (Carl Roth) for 10 min at room temperature. Cells were incubated for 1 h with 5% goat serum (Sigma–Aldrich) in PBS (Life Technologies, Carlsbad, CA, USA) as a blocking solution to prevent nonspecific antibody binding. The primary anti-PD-L1 antibody (rabbit mAb, clone E1L3N, #13684, CST) was diluted to 3 µg/mL in 3% BSA in PBS (Life Technologies) and incubated overnight at 4 °C. A rabbit monoclonal IgG antibody (#3900, CST) was used as an isotype control at the same concentration. For signal detection, slides were incubated for 10 min with EnVision™+ Dual Link System-HRP (DAKO, Agilent, Santa Clara, CA, USA). Visualization of immunoreactivity was performed using 3,3’-diaminobenzidine (DAB) tablets (Sigma–Aldrich). The samples were incubated for 30 min. The samples were then dehydrated by an ascending alcohol series and coverslipped with Entellan. Samples were digitally photographed in bright field illumination at 20-fold magnification under a microscope (Nikon Eclipse TE2000-U, Nikon, Minato, J).

### 4.9. Transient siRNA Knockdown

To decrease PD-L1 expression (KD), cells were transiently transfected with small interfering RNA (siRNA) directed against the human PD-L1 gene CD274. Dharmafect-1 (Thermo Scientific) was used as the transfection reagent according to the manufacturer’s instructions.

This siRNA was designed to reduce off-target effects by more than 90%. The siRNA consisted of a pool of 4 different siRNAs (the sequences are listed below in Table 1) so that elimination was guaranteed. Seed regions play a key role in causing off-target effects. To avoid these seed region caused off-target effects, special design filters were applied to eliminate frequent seed regions. Transfection was performed in 6-well plates (Corning, NY, USA). For reverse transfection, a cell suspension of 2 × 10^5^ cells was added to prepared transfection complexes. Transfection with 25 nM siRNA was carried out in DMEM growth medium without antibiotics for 72 h. The siGENOME Human CD274 siRNA SMARTpool (M-015836-01-0005, Horizon Discovery, Cambridge, UK) consisted of four siRNA target sequences. The ON-TARGETplus Non-Targeting Control Pool (D-001810-10-20, Horizon Discovery) was used as a control. The SiRNA sequences used for PD-L1 knockdown experiments are listed in Table 1. Three days after transfection, cells were relocated for optimal confluence and used for experiments.

### 4.10. Statistical Analysis

Statistical analyses were performed using GraphPad Prism 8 software (GraphPad Software, Inc.). All assays were performed in replicates, and the results are presented as means ± SD. Two-tailed Student’s *t*-test, ratio paired *t*-tests or multiple *t*-tests were used for comparison between groups. *p* ≤ 0.05 was considered as statistically significant.

## Figures and Tables

**Table 1:** SiRNA sequences used for PD-L1 knockdown experiments.

PD-L1 siRNA SMARTpool	Non-Targeting Control Pool
AGACCUGGCUGCACUAAUU (D-015836-03)	UGGUUUACAUGUCGACUAA
UGAAAGGACUCACUUGGUA (D-015836-01)	UGGUUUACAUGUUGUGUGA
CAUAGUAGCUACAGACAGA (D-015836-02)	UGGUUUACAUGUUUUCUGA
GGACCUAUAUGUGGUAGAG (D-015836-04)	UGGUUUACAUGUUUUCCUA

## Supplementary Materials

The following are available online at https://www.mdpi.com/article/10.3390/ijms222313087/s1.

## Abbreviations

APC	antigen-presenting cell
BSA	bovine serum albumin
CD	cluster of differentiation
CDK	cyclin-dependent kinase
CST	Cell Signaling Technologies
DAB 3,3’	diaminobenzidine
DDRD	DNA damage response is deficient
DMEM	Dulbecco’s modified Eagle’s medium
DMSO	dimethyl sulfoxide
EDTA	ethylenediaminetetraacetic acid
FACS	fluorescence-activated cell scanning
FCS	fetal calf serum
HNSCC	head and neck squamous cell carcinoma
HPV	human papillomavirus
HRP	horseradish peroxidase
IC_50_	half maximal inhibitory concentration
IgG	immunoglobulin G
KD	knockdown
M phase	mitosis phase
mAb	monoclonal antibody
MDSC	myeloid-derived suppressor cells
MEF	mouse embryonic fibroblast
MTT	3-(4,5-dimethylthiazol-2-yl)-2,5-diphenyl tetrazolium bromide
nM	millimolar
ns	not significant
NT	non-targeting
ORR	overall response rate
pAb	polyclonal antibody
PBS	phosphate-buffered saline
PCI	Pittsburgh Cancer Institute
PCR	polymerase chain reaction
PD-1	programmed cell death protein-1
PD-L1	programmed cell death ligand-1
PE	phycoerythrin
PFA	paraformaldehyde
RB	retinoblastoma
RT	room temperature
S phase	synthesis phase
SD	standard deviation
SDS-PAGE	sodium dodecyl sulfate polyacrylamide gel electrophoresis
siRNA	small interfering ribonucleic acid
SPOP	speckle-type POZ protein
TIL	tumor-infiltrating lymphocyte
Tregs	regulatory T cells
WAPL	wing apart-like
WB	Western blot

## References

[1] World Health Organization: Regional Office for Europe (2020). World Cancer Report: Cancer Research for Cancer Prevention.

[2] Santuray R.T., Johnson D.E., Grandis J.R. (2018). New Therapies in Head and Neck Cancer. Trends Cancer.

[3] Keir M.E., Butte M.J., Freeman G.J., Sharpe A.H. (2008). PD-1 and Its Ligands in Tolerance and Immunity. Annu. Rev. Immunol..

[4] Topalian S.L., Drake C.G., Pardoll D.M. (2012). Targeting the PD-1/B7-H1(PD-L1) Pathway to Activate Anti-Tumor Immunity. Curr. Opin. Immunol..

[5] Chen J., Jiang C.C., Jin L., Zhang X.D. (2016). Regulation of PD-L1: A Novel Role of pro-Survival Signalling in Cancer. Ann. Oncol..

[6] Gevensleben H., Dietrich D., Golletz C., Steiner S., Jung M., Thiesler T., Majores M., Stein J., Uhl B., Muller S. (2016). The Immune Checkpoint Regulator PD-L1 Is Highly Expressed in Aggressive Primary Prostate Cancer. Clin. Cancer Res..

[7] Lin Y.-M., Sung W.-W., Hsieh M.-J., Tsai S.-C., Lai H.-W., Yang S.-M., Shen K.-H., Chen M.-K., Lee H., Yeh K.-T. (2015). High PD-L1 Expression Correlates with Metastasis and Poor Prognosis in Oral Squamous Cell Carcinoma. PLoS ONE.

[8] Straub M., Drecoll E., Pfarr N., Weichert W., Langer R., Hapfelmeier A., Götz C., Wolff K.-D., Kolk A., Specht K. (2016). *CD274/PD-L1* Gene Amplification and PD-L1 Protein Expression Are Common Events in Squamous Cell Carcinoma of the Oral Cavity. Oncotarget.

[9] Wu P., Wu D., Li L., Chai Y., Huang J. (2015). PD-L1 and Survival in Solid Tumors: A Meta-Analysis. PLoS ONE.

[10] Ferris R.L., Blumenschein G., Fayette J., Guigay J., Colevas A.D., Licitra L., Harrington K., Kasper S., Vokes E.E., Even C. (2016). Nivolumab for Recurrent Squamous-Cell Carcinoma of the Head and Neck. N. Engl. J. Med..

[11] Seiwert T.Y., Burtness B., Mehra R., Weiss J., Berger R., Eder J.P., Heath K., McClanahan T., Lunceford J., Gause C. (2016). Safety and Clinical Activity of Pembrolizumab for Treatment of Recurrent or Metastatic Squamous Cell Carcinoma of the Head and Neck (KEYNOTE-012): An Open-Label, Multicentre, Phase 1b Trial. Lancet Oncol..

[12] Gotwals P., Cameron S., Cipolletta D., Cremasco V., Crystal A., Hewes B., Mueller B., Quaratino S., Sabatos-Peyton C., Petruzzelli L. (2017). Prospects for Combining Targeted and Conventional Cancer Therapy with Immunotherapy. Nat. Rev. Cancer.

[13] Sharma P., Allison J.P. (2015). The Future of Immune Checkpoint Therapy. Science.

[14] Zhou K.I., Peterson B., Serritella A., Thomas J., Reizine N., Moya S., Tan C., Wang Y., Catenacci D.V.T. (2020). Spatial and Temporal Heterogeneity of PD-L1 Expression and Tumor Mutational Burden in Gastroesophageal Adenocarcinoma at Baseline Diagnosis and after Chemotherapy. Clin. Cancer Res..

[15] Herbst R.S., Soria J.-C., Kowanetz M., Fine G.D., Hamid O., Gordon M.S., Sosman J.A., McDermott D.F., Powderly J.D., Gettinger S.N. (2014). Predictive Correlates of Response to the Anti-PD-L1 Antibody MPDL3280A in Cancer Patients. Nature.

[16] Iwai Y., Ishida M., Tanaka Y., Okazaki T., Honjo T., Minato N. (2002). Involvement of PD-L1 on Tumor Cells in the Escape from Host Immune System and Tumor Immunotherapy by PD-L1 Blockade. Proc. Natl. Acad. Sci. USA.

[17] Yu J., Qin B., Moyer A.M., Nowsheen S., Tu X., Dong H., Boughey J.C., Goetz M.P., Weinshilboum R., Lou Z. (2020). Regulation of Sister Chromatid Cohesion by Nuclear PD-L1. Cell Res..

[18] Zhang J., Bu X., Wang H., Zhu Y., Geng Y., Nihira N.T., Tan Y., Ci Y., Wu F., Dai X. (2018). Cyclin D-CDK4 Kinase Destabilizes PD-L1 via Cullin 3-SPOP to Control Cancer Immune Surveillance. Nature.

[19] Boussiotis V.A. (2016). Molecular and Biochemical Aspects of the PD-1 Checkpoint Pathway. N. Engl. J. Med..

[20] Gato-Cañas M., Zuazo M., Arasanz H., Ibañez-Vea M., Lorenzo L., Fernandez-Hinojal G., Vera R., Smerdou C., Martisova E., Arozarena I. (2017). PDL1 Signals through Conserved Sequence Motifs to Overcome Interferon-Mediated Cytotoxicity. Cell Rep..

[21] Clark C.A., Gupta H.B., Sareddy G., Pandeswara S., Lao S., Yuan B., Drerup J.M., Padron A., Conejo-Garcia J., Murthy K. (2016). Tumor-Intrinsic PD-L1 Signals Regulate Cell Growth, Pathogenesis, and Autophagy in Ovarian Cancer and Melanoma. Cancer Res..

[22] Chen L., Xiong Y., Li J., Zheng X., Zhou Q., Turner A., Wu C., Lu B., Jiang J. (2017). PD-L1 Expression Promotes Epithelial to Mesenchymal Transition in Human Esophageal Cancer. Cell. Physiol. Biochem..

[23] Eichberger J., Schulz D., Pscheidl K., Fiedler M., Reichert T.E., Bauer R.J., Ettl T. (2020). PD-L1 Influences Cell Spreading, Migration and Invasion in Head and Neck Cancer Cells. Int. J. Mol. Sci..

[24] Schulz D., Streller M., Piendl G., Brockhoff G., Reichert T.E., Menevse A.N., Beckhove P., Hautmann M.G., Bauer R.J., Ettl T. (2020). Differential Localization of PD-L1 and Akt-1 Involvement in Radioresistant and Radiosensitive Cell Lines of Head and Neck Squamous Cell Carcinoma. Carcinogenesis.

[25] Schulz D., Stancev I., Sorrentino A., Menevse A.-N., Beckhove P., Brockhoff G., Hautmann M.G., Reichert T.E., Bauer R.J., Ettl T. (2018). Increased PD-L1 Expression in Radioresistant HNSCC Cell Lines after Irradiation Affects Cell Proliferation Due to Inactivation of GSK-3beta. Oncotarget.

[26] Cavalieri S., Rivoltini L., Bergamini C., Locati L.D., Licitra L., Bossi P. (2018). Immuno-Oncology in Head and Neck Squamous Cell Cancers: News from Clinical Trials, Emerging Predictive Factors and Unmet Needs. Cancer Treat. Rev..

[27] Stevanović S., Pasetto A., Helman S.R., Gartner J.J., Prickett T.D., Howie B., Robins H.S., Robbins P.F., Klebanoff C.A., Rosenberg S.A. (2017). Landscape of Immunogenic Tumor Antigens in Successful Immunotherapy of Virally Induced Epithelial Cancer. Science.

[28] Mandal R., Şenbabaoğlu Y., Desrichard A., Havel J.J., Dalin M.G., Riaz N., Lee K.-W., Ganly I., Hakimi A.A., Chan T.A. (2016). The Head and Neck Cancer Immune Landscape and Its Immunotherapeutic Implications. JCI Insight.

[29] Han L., Liu F., Li R., Li Z., Chen X., Zhou Z., Zhang X., Hu T., Zhang Y., Young K. (2014). Role of Programmed Death Ligands in Effective T-Cell Interactions in Extranodal Natural Killer/T-Cell Lymphoma. Oncol. Lett..

[30] McGee H.S., Yagita H., Shao Z., Agrawal D.K. (2010). Programmed Death-1 Antibody Blocks Therapeutic Effects of T-Regulatory Cells in Cockroach Antigen-Induced Allergic Asthma. Am. J. Respir. Cell Mol. Biol..

[31] Ferris R.L. (2015). Immunology and Immunotherapy of Head and Neck Cancer. J. Clin. Oncol..

[32] Zandberg D.P., Strome S.E. (2014). The Role of the PD-L1:PD-1 Pathway in Squamous Cell Carcinoma of the Head and Neck. Oral Oncol..

[33] Yu G.-T., Bu L.-L., Huang C.-F., Zhang W.-F., Chen W.-J., Gutkind J.S., Kulkarni A.B., Sun Z.-J. (2015). PD-1 Blockade Attenuates Immunosuppressive Myeloid Cells Due to Inhibition of CD47/SIRPα Axis in HPV Negative Head and Neck Squamous Cell Carcinoma. Oncotarget.

[34] Chow L.Q.M., Haddad R., Gupta S., Mahipal A., Mehra R., Tahara M., Berger R., Eder J.P., Burtness B., Lee S.-H. (2016). Antitumor Activity of Pembrolizumab in Biomarker-Unselected Patients With Recurrent and/or Metastatic Head and Neck Squamous Cell Carcinoma: Results From the Phase Ib KEYNOTE-012 Expansion Cohort. J. Clin. Oncol..

[35] Chen D.S., Mellman I. (2017). Elements of Cancer Immunity and the Cancer-Immune Set Point. Nature.

[36] Brahmer J., Reckamp K.L., Baas P., Crinò L., Eberhardt W.E.E., Poddubskaya E., Antonia S., Pluzanski A., Vokes E.E., Holgado E. (2015). Nivolumab versus Docetaxel in Advanced Squamous-Cell Non-Small-Cell Lung Cancer. N. Engl. J. Med..

[37] Weber J.S., D’Angelo S.P., Minor D., Hodi F.S., Gutzmer R., Neyns B., Hoeller C., Khushalani N.I., Miller W.H., Lao C.D. (2015). Nivolumab versus Chemotherapy in Patients with Advanced Melanoma Who Progressed after Anti-CTLA-4 Treatment (CheckMate 037): A Randomised, Controlled, Open-Label, Phase 3 Trial. Lancet Oncol..

[38] Fiedler M., Schulz D., Piendl G., Brockhoff G., Eichberger J., Menevse A.-N., Beckhove P., Hautmann M., Reichert T.E., Ettl T. (2020). Buparlisib Modulates PD-L1 Expression in Head and Neck Squamous Cell Carcinoma Cell Lines. Exp. Cell Res..

[39] Tufano M., D’Arrigo P., D’Agostino M., Giordano C., Marrone L., Cesaro E., Romano M.F., Romano S. (2021). PD-L1 Expression Fluctuates Concurrently with Cyclin D in Glioblastoma Cells. Cells.

[40] Jin X., Ding D., Yan Y., Li H., Wang B., Ma L., Ye Z., Ma T., Wu Q., Rodrigues D.N. (2019). Phosphorylated RB Promotes Cancer Immunity by Inhibiting NF-ΚB Activation and PD-L1 Expression. Mol. Cell.

[41] Ullah M., Aoudjeghout W., Pimpie C., Pocard M., Mirshahi M. (2020). Mitosis in Cancer Cell Increases Immune Resistance via High Expression of HLA-G and PD-L1. Cancers.

[42] Zhang X., Huang X., Xu J., Li E., Lao M., Tang T., Zhang G., Guo C., Zhang X., Chen W. (2021). NEK2 Inhibition Triggers Anti-Pancreatic Cancer Immunity by Targeting PD-L1. Nat. Commun..

[43] Parkes E.E., Walker S.M., Taggart L.E., McCabe N., Knight L.A., Wilkinson R., McCloskey K.D., Buckley N.E., Savage K.I., Salto-Tellez M. (2017). Activation of STING-Dependent Innate Immune Signaling By S-Phase-Specific DNA Damage in Breast Cancer. J. Natl. Cancer Inst..

[44] Zuo H.X., Jin Y., Wang Z., Li M.Y., Zhang Z.H., Wang J.Y., Xing Y., Ri M.H., Jin C.H., Xu G.H. (2020). Curcumol Inhibits the Expression of Programmed Cell Death-Ligand 1 through Crosstalk between Hypoxia-Inducible Factor-1α and STAT3 (T705) Signaling Pathways in Hepatic Cancer. J. Ethnopharmacol..

[45] Heo D.S., Snyderman C., Gollin S.M., Pan S., Walker E., Deka R., Barnes E.L., Johnson J.T., Herberman R.B., Whiteside T.L. (1989). Biology, Cytogenetics, and Sensitivity to Immunological Effector Cells of New Head and Neck Squamous Cell Carcinoma Lines. Cancer Res..

[46] Lin C.J., Grandis J.R., Carey T.E., Gollin S.M., Whiteside T.L., Koch W.M., Ferris R.L., Lai S.Y. (2007). Head and Neck Squamous Cell Carcinoma Cell Lines: Established Models and Rationale for Selection. Head Neck.

