# Supplementary material for: Differential Expression of PD-L1 during Cell Cycle Progression of Head and Neck Squamous Cell Carcinoma
Source: Int J Mol Sci. 2021 Dec 3;22(23):13087. doi: 10.3390/ijms222313087 (PMC8658507; doi:10.3390/ijms222313087)
Supplement: Supplementary file 1 [file ijms-22-13087-s001.zip › ijms-1472529-supplementary.pdf]

Supplement

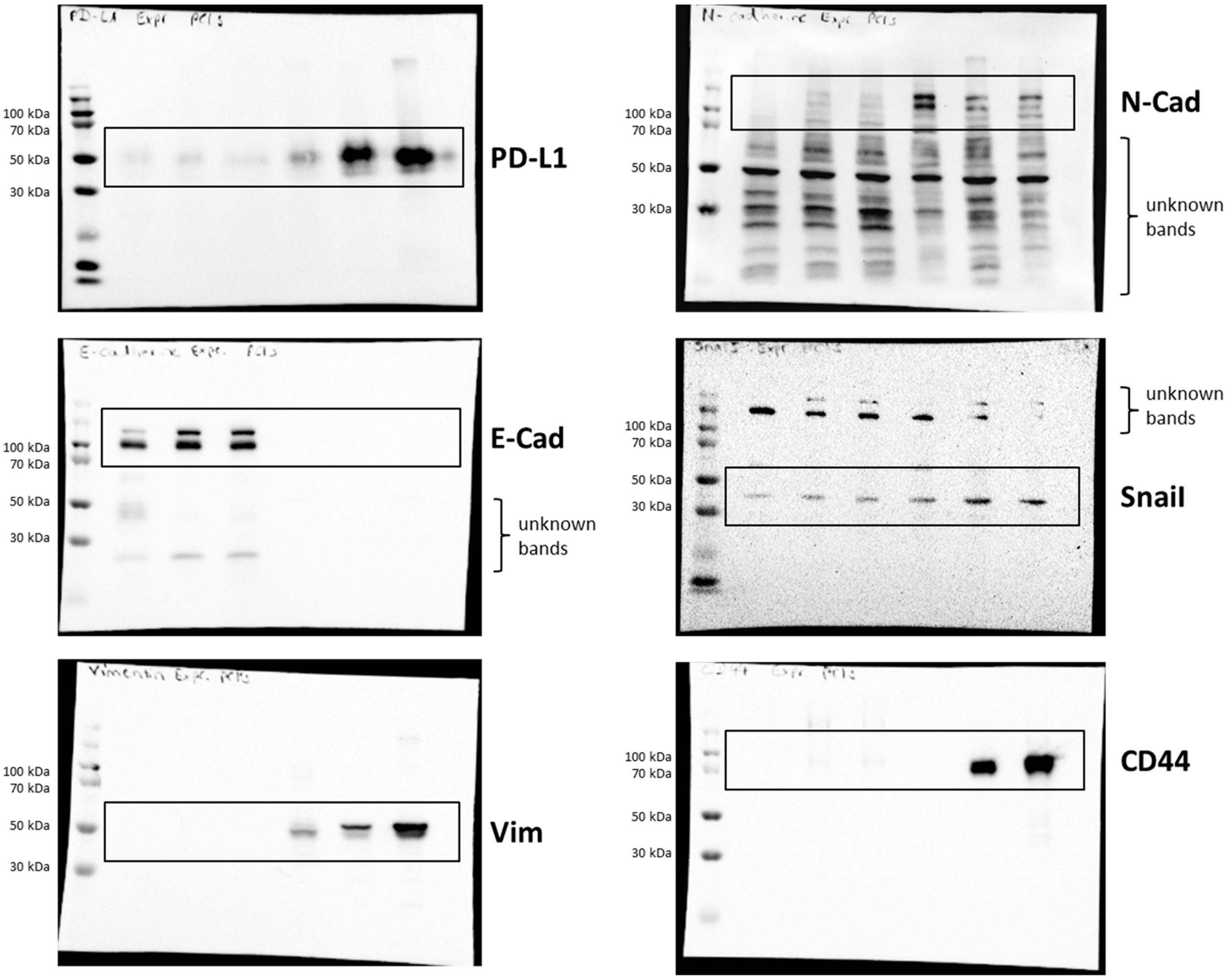

Supplementary Materials Figure S1. Original Western blots for characterization of HNSCC cell lines. Thirty micrograms of cell lysate were used from each sample. PD-L1, E-cadherin (E-Cad), vimentin (Vim), N-cadherin N-Cad, Snai1, and CD44 were detected with specific antibodies.

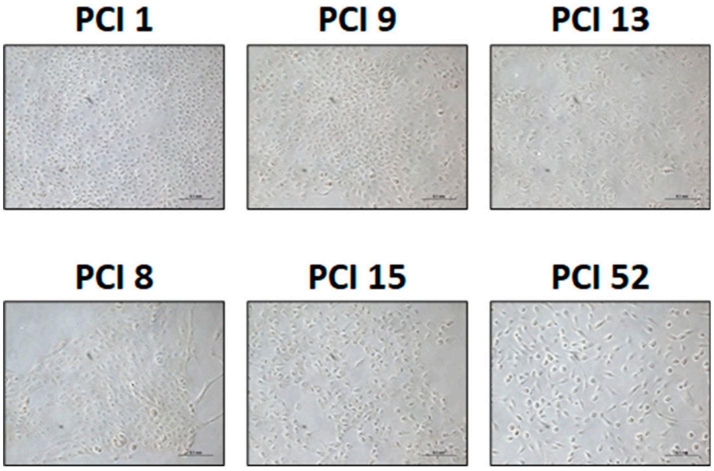

Supplementary Materials Figure S2. Control for immunocytochemical staining of PD-L1 with DAB. Instead of the specific antibody, cells here were incubated with an IgG isotype control to demonstrate the specificity of PD-L1 staining. Cells were pretreated with DMSO and, therefore, consisted of a mixed cell population with randomly distributed cells at different cell cycle phases. Representative images of the HNSCC cell lines PCI 1, 9, 13, 8, 15, and 52 are shown at 20-fold magnification. The scale represents 0.1 mm.

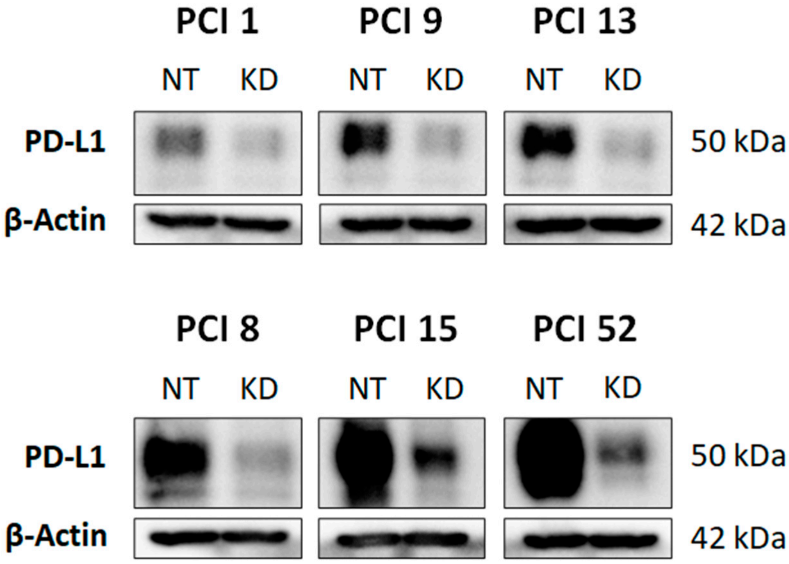

Supplementary Materials Figure S3. SiRNA transfection control. WB analysis shows a decrease in PD-L1 expression after siRNA knockdown (KD) compared to non-targeting siRNA control (NT) in HNSCC cell lines PCI 1, 9, 13, 8, 15, and 52. For each sample, 30 μg total protein lysate was used. β-actin served as a loading control.

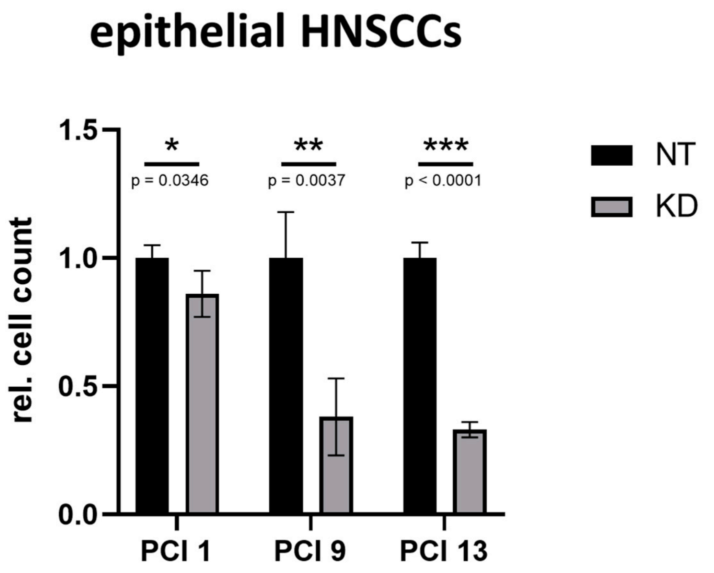

Supplementary Materials Figure S4. Proliferation after PD-L1 KD in epithelial HNSCCs. Semiquantitative analysis of the MTT proliferation assay. Cell counts after siRNA knockdown (KD) were compared with siRNA control (NT) in epithelial HNSCC cell lines PCI 1, 9, and 13. NT control of each cell line was used as a reference. Results are expressed as means ± SD (standard deviation). Multiple *t*-test (* = $p < 0.05$; ** = $p < 0.01$; *** = $p < 0.001$). $n = 4$.
